# Plant sterol hyperabsorption caused by uncontrolled diabetes in a patient with a heterozygous *ABCG5* variant

**DOI:** 10.1111/jdi.13874

**Published:** 2022-07-21

**Authors:** Masashi Hasebe, Yorihiro Iwasaki, Yamato Keidai, Kanako Iwasaki, Sachiko Honjo, Akihiro Hamasaki

**Affiliations:** ^1^ Department of Diabetes and Endocrinology Tazuke Kofukai Medical Research Institute Kitano Hospital Osaka Japan; ^2^ Endocrine Unit Department of Medicine Massachusetts General Hospital and Harvard Medical School Boston Massachusetts USA; ^3^ Department of Diabetes, Endocrinology and Nutrition Kyoto University Graduate School of Medicine Kyoto Japan

**Keywords:** *ABCG5*, Plant sterols, Type 1 diabetes

## Abstract

Plant sterol intake is widely recommended for patients with cardiovascular risk factors based on the inhibitory effect on intestinal cholesterol absorption. Although plant sterols, once absorbed, can promote atherosclerosis, their intake is believed to be safe because of poor absorption, except in rare hyperabsorbers with homozygous *ABCG5/8* mutations. We report a case of new‐onset type 1 diabetes accompanied by hypercholesterolemia. At the initial presentation with diabetic ketoacidosis, the patient showed marked hypercholesterolemia. Whole‐exome sequencing revealed a heterozygous pathogenic variant in *ABCG5* (p.R419H). The initial serum plant sterol levels were markedly high (sitosterol 32.5 μg/mL, campesterol 66.0 μg/mL), close to the range observed in patients with homozygous *ABCG5/8* mutations, which were largely reduced by insulin treatment without ezetimibe. The addition of ezetimibe normalized plant sterol levels. These findings provide the first evidence that uncontrolled diabetes plays a causal role in the pathogenesis of phytosterolemia.

## Graphical abstract

Plant sterol intake is widely recommended in patients with cardiovascular risk factors based on the inhibitory effect on intestinal cholesterol absorption, except for rare hyperabsorbers with homozygous ABCG5/8 mutations. We report a case of new‐onset type 1 diabetes accompanied by hypercholesterolemia and phytosterolemia, which were improved by insulin and ezetimibe. In this report, we show an unrecognized risk of plant sterol hyperabsorption in patients with uncontrolled diabetes through comprehensive genetic and biochemical analyses of the index patient and family members, highlighting an overlooked potential pitfall when applying the current recommendation for dietary plant sterol intake.

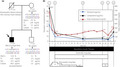

## INTRODUCTION

Plant sterols (or phytosterols), such as sitosterol and campesterol, inhibit intestinal cholesterol absorption because of their structural similarity to cholesterol. Dietary plant sterols taken up by the intestinal mucosa are largely excreted into the feces by adenosine triphosphate‐binding cassette subfamily G members 5 (ABCG5) and 8 (ABCG8).^1^ Based on their cholesterol‐lowering effect, plant sterol consumption has been widely recommended for patients at risk of cardiovascular disease, including those with diabetes.^2^ However, once absorbed, plant sterols can promote atherosclerosis, which is observed in patients with sitosterolemia caused by biallelic loss‐of‐function mutations in *ABCG5/8* genes.^1^ In patients with diabetes, although some studies suggested increased plant sterol absorption,^3^, ^4^ there is no direct evidence of its changes in response to the treatment of hyperglycemia. Therefore, it is unclear whether the recommendation to increase plant sterol consumption is safe for every patient with diabetes. We herein report a patient with a heterozygous loss of function *ABCG5* mutation, who showed marked phytosterolemia and hypercholesterolemia at the onset of type 1 diabetes. Insulin treatment substantially decreased his serum plant sterol levels, and the addition of ezetimibe further reduced his serum plant sterol levels to the normal range. In the present study, we provide the first direct evidence that uncontrolled diabetes has a causal role in phytosterolemia.

## CASE REPORT

A 21‐year‐old Japanese man (height 174.3 cm, weight 54.8 kg and body mass index 18.0 kg/m^2^) without remarkable medical history was admitted to Kitano Hospital, Osaka, Japan, complaining of polydipsia, polyuria and unintentional bodyweight loss for 2 months. At the presentation, physical examination revealed tachycardia, dry oral mucous membranes and deep respiration, but no physical findings characteristic of familial hypercholesterolemia, such as xanthomas and corneal rings. Based on ketonuria, metabolic acidosis and hyperglycemia, he was diagnosed with diabetic ketoacidosis (Table 1). Given the presence of anti‐glutamic acid decarboxylase antibody and severely blunted serum C‐peptide response during the glucagon stimulation test, we diagnosed the patient as acute‐onset type 1 diabetes. Intensive insulin therapy was initiated immediately after admission, and his diabetic ketoacidosis resolved on hospital day 2. The patient was discharged on hospital day 19 with insulin degludec and insulin aspart for routine glycemic control, and insulin treatment drastically reduced his glycated hemoglobin level from 15.8% at the baseline to 6.2% at 2 months.

**Table 1:** Laboratory data of the patient at the initial presentation

Variable	Result	Reference	Variable	Result	Reference
Peripheral blood			Diabetes		
White blood cells (/μL)	14,100	3,300–8,600	Plasma glucose (mg/dL)	433	73–109
Neutrophil (%)	75.8	41.7–33.7	HbA1c (%)	15.8	4.6–6.2
Eosinophil (%)	0.0	0.7–8.1	C‐peptide (ng/mL)	0.36	0.74–3.48
Red blood cells (×10^4^/μL)	606	435–555	Anti‐GADAb (U/mL)	6.5	<5.0
Hemoglobin (g/dL)	18.6	13.7–16.8	Anti‐InsulinAb (U/mL)	<0.4	<0.4
Hematocrit (%)	58.1	40.7–50.1	Anti‐IA‐2Ab (U/mL)	<0.6	<0.6
Platelets (×10^4^/μL)	18.8	15.8	Anti‐ZnT8Ab (U/mL)	<15.0	<15.0
Blood biochemistry			Glucagon stimulation test		
Total protein (g/dL)	8.4	6.6–8.1	C‐peptide (0 min) (ng/mL)	0.17	
Albumin (g/dL)	5.5	4.1–5.1	C‐peptide (6 min) (ng/mL)	0.38	
AST (U/L)	18	13–30	Thyroid marker		
ALT (U/L)	20	10–42	FT4 (ng/dL)	0.96	0.95–1.74
LDH (U/L)	189	124–222	FT3 (pg/mL)	1.51	2.13–4.07
ALP (U/L)	341	106–322	TSH (μU/mL)	1.610	0.34–3.88
γ‐GTP (U/L)	44	13–64	Anti‐TPOAb (IU/mL)	<9	<9
BUN (mg/dL)	15.6	8.0–20.0	Anti‐TgAb (IU/mL)	<10	<10
Creatinine (mg/dL)	1.04	0.65–1.07	Arterial blood gas analysis		
Uric acid (mg/dL)	6.6	3.7–7.8	pH	7.081	7.35–7.45
Creatinine kinase (U/L)	348	59–248	PaCO_2_ (mmHg)	7.1	35–45
Sodium (mmol/L)	134	138–145	PaO_2_ (mmHg)	136.0	80–100
Potassium (mmol/L)	4.6	3.6–4.8	${\mathrm{HCO}}_{3}^{-}$ (mmol/L)	2.0	22–28
Chloride (mmol/L)	102	101–108	Base excess (mmol/L)	−30.5	−2.0–2.0
Total cholesterol (mg/dL)	670	142–248	Urinary test		
C‐reactive protein (mg/dL)	0.05	0–0.14	Urinary pH	5.5	4.5–7.5
Total ketone body (μmol/L)	14,796	<130	Urinary protein	2+	–
Acetoacetate (μmol/L)	2,235	<55	Urinary glucose	3+	–
β‐hydroxybuterate (μmol/L)	12,561	<85	Urinary ketone body	3+	–

ALP, alkaline phosphatase; ALT, alanine aminotransferase; Anti‐GADAb, anti‐glutamic acid decarboxylase antibody; Anti‐IA‐2 antibody, anti‐insulinoma‐associated protein‐2 antibody; Anti‐InsulinAb, anti‐insulin antibody; Anti‐TgAb, anti‐thyroglobulin antibody; Anti‐TPOAb, anti‐thyroid peroxidase antibody; Anti‐ZnT8Ab, anti‐zinc transporter protein 8 antibody; AST, aspartate aminotransferase; BUN, blood urea nitrogen; FT3, free triiodothyronine; FT4, free thyroxine; HbA1c, glycated hemoglobin; LDH, lactate dehydrogenase; γ‐GTP, γ‐glutamyltranspeptidase.

**Figure 1:**
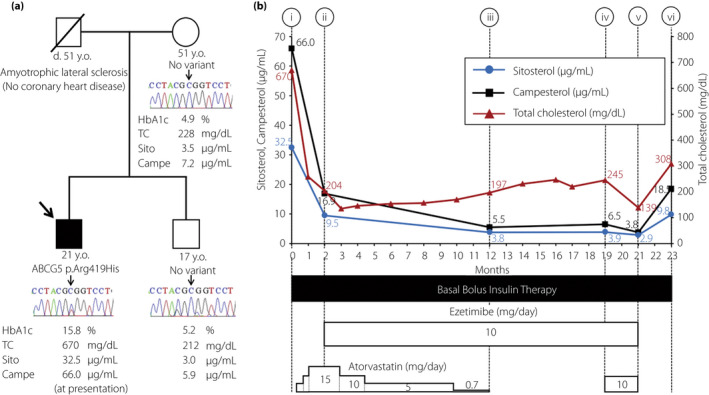
(a) Sanger sequencing results of *ABCG5* gene (chromatogram) and laboratory results (glycated hemoglobin [HbA1c], total cholesterol [TC], sitosterol [Sito], and campesterol [Campe]) of the patient and family members. (b) The clinical course of serum sitosterol (blue circles), campesterol (black squares) and TC (red triangles) levels. Time points (i) through (vi) correspond to those in Table 1. [Colour figure can be viewed at wileyonlinelibrary.com]

Notably, the initial biochemical evaluation showed an extremely high level of serum total cholesterol (670 mg/dL). Laboratory test results at the presentation are summarized in Table 1 and Figure S1. The marked hypercholesterolemia at the presentation made us consider the possibility of familial hypercholesterolemia, and we carried out whole‐exome sequencing of the patient. The whole‐exome sequencing of the patient's genome revealed a heterozygous variant in *ABCG5* (c.1256G > A or p.Arg419His), which was confirmed by Sanger sequencing (Figure 1a). The variant was reported to be pathogenic.^5^ There were no pathogenic variants in *LDLR*, *PCSK9*, *APOB*, *LDLRAP*, *CYP27A1* and *ABCG8* in the patient's genome. Based on these results, we measured serum plant sterol levels of the patient at the initial presentation, which showed marked elevation of both sitosterol and campesterol (32.5 μg/mL and 66.0 μg/mL, respectively) close to the range of patients with homozygous *ABCG5/8* mutations.^6^ His mother and brother did not have the *ABCG5* variant, and showed normal serum plant sterols levels (Figure 1a). We were unable to obtain his father's lipid profile and genomic DNA, because his father had already died of amyotrophic lateral sclerosis. Therefore, we could not determine whether the *ABCG5* variant is paternally derived or de novo. All genetic analyses of the patient and his family members were approved by the local ethics committee of Kitano Hospital (No. 180400601, approved on 17 April 2018). Intriguingly, after improved glycemic control with insulin, plant sterol concentrations were decreased to approximately 25% of initial levels without ezetimibe (Figure 1b). At that point, ezetimibe was initiated considering the possibility of increased plant sterol absorption. In addition, although a detailed dietary interview showed that the patient had not consumed excess plant sterol‐enriched foods before the onset of type 1 diabetes, we instructed the patient to avoid dietary intake of plant sterols. Thereafter, serum sitosterol and campesterol levels were normalized. To further confirm the effects of ezetimibe or atorvastatin, we serially measured serum plant sterols after the sequential discontinuation of each medication. The results showed elevation of plant sterol levels only after discontinuation of ezetimibe, even under optimal glycemic control. The clinical course of glycemic and lipid profiles of the patient are summarized in Table 2 and Figure 1b.

**Table 2:** The clinical course of glycemic and lipid profiles of the patient

	(i)		(ii)	(iii)	(iv)	(v)	(vi)
Time from presentation (months)	0 (hospital day 1)	0 (hospital day 4)	2	12	19	21	23
Total cholesterol (mg/dL)	670	403	204	197	245	139	308
Triglycerides (mg/dL)	–	138	71	105	183	83	216
LDL‐C (mg/dL)	–	326	132	110	56	63	228
HDL‐C (mg/dL)	–	44	57	68	160	61	53
Sitosterol (μg/mL)	32.5	–	9.5	3.8	3.9	2.9	9.8
Campesterol (μg/mL)	66.0	–	16.9	5.5	6.5	3.8	18.5
Lathosterol (μg/mL)	1.6	–	0.2	1.0	1.8	0.6	1.6
Apolipoprotein A‐I (mg/dL)	–	110	–	–	–	–	–
Apolipoprotein B (mg/dL)	–	222	–	–	–	–	–
Apolipoprotein C–II (mg/dL)	–	9.0	–	–	–	–	–
Apolipoprotein E (mg/dL)	–	6.3	–	–	–	–	–
HbA1c (%)	15.8	–	6.2	6.0	6.3	6.6	6.4
Plasma glucose (mg/dL)	433	274	79	116	131	167	117

The time points (i) through (vi) in the top row correspond to those in the graph in Figure 1b. Reference intervals: sitosterol (μg/mL) 0.99–3.88; campesterol (μg/mL) 2.14–7.43; lathosterol (μg/mL) 0.77–3.60; apoliporotein A‐I (mg/dL) 119–155; apolipoprotein B (mg/dL) 73–109; apolipoprotein C‐II (mg/dL) 1.8–4.6; apolipoprotein E (mg/dL) 1.7–4.3. HbA1c, glycated hemoglobin; HDL‐C, high‐density‐lipoprotein cholesterol; LDL‐C, low‐density‐lipoprotein cholesterol.

## DISCUSSION

In the present study, we characterized marked phytosterolemia in a patient with uncontrolled diabetes who has a heterozygous *ABCG5* mutation. The current findings have two important implications: (i) pathogenesis of phytosterolemia in uncontrolled diabetes; and (ii) the potential risk of plant sterol intake in patients with uncontrolled diabetes and/or heterozygous *ABCG5/8* mutations.

Mechanistically, dynamic changes in serum plant sterol levels might reflect both insulin deficiency (or hyperglycemia) and the heterozygous *ABCG5* mutation. Initial marked phytosterolemia and its substantial reversal by glycemic control without ezetimibe indicate a major causal role of uncontrolled diabetes in phytosterolemia. This notion is supported by previous studies showing elevated serum plant sterol levels in patients with type 1 diabetes.^3^, ^7^ The present data provide the first direct evidence of increased plant sterol levels in uncontrolled diabetes and a dramatic decrease in response to glycemic control. Although the precise mechanisms of plant sterol hyperabsorption in diabetes remain uncertain, a study using a streptozotocin‐induced rat diabetes murine model suggested that reduced *ABCG5/8* mRNA expression in both liver and intestine leads to increased cholesterol absorption.^8^ Another study also showed that *ABCG5/8* mRNA expression levels in duodenal biopsies were lower in patients with type 2 diabetes compared with healthy individuals.^4^ Taken together with the association between hyperinsulinemia and decreased cholesterol absorption,^9^ insulin deficiency might contribute to increased plant sterol absorption through reduced *ABCG5/8* expression.

On the other hand, normalization of serum plant sterols was achieved only by adding ezetimibe, suggesting that the heterozygous *ABCG5* mutation predisposed the patient to elevated basal plant sterol levels. The extent of plant sterol absorption in heterozygous *ABCG5*/*8* mutation carriers remains controversial.^6^, ^10^ Recent studies showed that heterozygous carriers have significantly higher serum plant sterol and cholesterol levels compared to healthy individuals.^6^ The present case also suggests that *ABCG5/8* heterozygous carriers are potential ‘hyperabsorbers’ for plant sterols.

Altogether, it seems plausible that the initial marked phytosterolemia in the current case (serum sitosterol and campesterol, 32.5 and 66.0 μg/mL, respectively) resulted from the coincidence of two factors that potentially deteriorate *ABCG5* function and increase plant sterol accumulation: the development of type 1 diabetes and the loss of function heterozygous mutation in *ABCG5*. Insulin treatment without ezetimibe largely decreased the levels of serum plant sterols (serum sitosterol and campesterol, 9.5 and 16.9 μg/mL, respectively), which conceivably reflected the restoration of *ABCG5/8* expression.^4^, ^8^ In addition, the normalization of serum plant sterol levels was achieved only with ezetimibe under optimal glycemic control, as the heterozygous *ABCG5* mutation contributed to slightly increased absorption of plant sterols.^6^

Due to their cholesterol‐lowering effect, plant sterol intake has been widely recommended to reduce cardiovascular risk among patients both with and without diabetes.^2^ Meanwhile, if absorbed from the intestine, plant sterols can promote atherosclerosis, which is suggested by the studies of the patients with sitosterolemia^1^ and the general population.^11^ Based on these observations and the present findings, the current recommendation of plant sterol intake should be carefully applied to patients with uncontrolled diabetes. It is noteworthy that the estimated incidence of deleterious *ABCG5/8* mutations is one in 220 individuals,^12^ indicating that a substantial number of patients with diabetes would be potential plant sterol ‘hyperabsorbers’. As most heterozygous carriers present with hypercholesterolemia, clinicians should be cautious in dietary guidance and drug choice for patients with diabetes and hypercholesterolemia. Plant sterol measurement can be helpful in order not to overlook ‘hyperabsorbers’.

## DISCLOSURE

The authors declare no conflict of interest.

Approval of the research protocol: All clinical and genetic analyses were approved by the local ethics committee of Kitano Hospital (No. 180400601, approved on 17 April 2018).

Informed consent: Written informed consent was obtained from the patient and family members for the genetic testing and for the publication of this article.

Registry and the registration no. of the study/trial: N/A.

Animal studies: N/A.

## Supporting information


**Figure S1** **|** Serum lipoprotein fractions measured by electrophoresis on polyacrylamide gel on hospital day 4.
